# A rare presentation of glycogenic acanthosis on the lips: A case report

**DOI:** 10.1177/2050313X241307124

**Published:** 2024-12-24

**Authors:** Abdullah Ramadhan, Erin Chapman, Eunice Y Chow

**Affiliations:** 1 Division of Dermatology, Department of Medicine, University of Alberta, Edmonton, AB, Canada; 2 Department of Laboratory Medicine and Pathology, University of Alberta, Edmonton, AB, Canada

**Keywords:** Glycogenic acanthosis, lip lesions, white papules, esophageal GA

## Abstract

Glycogenic acanthosis (GA) is a benign, asymptomatic condition commonly found in the lower third of the esophagus. This case report presents a unique occurrence of GA on the lips of a 55-year-old male smoker, who exhibited asymptomatic white lesions on his upper and lower lips for many years. Physical examination revealed multiple white papules on the wet-dry vermilion border of the lips. Biopsies confirmed the diagnosis of GA, showing acanthosis, hyperkeratosis, and glycogenesis without dysplasia. The rarity of GA on the lips underscores the need to consider it in the differential diagnosis of white papules on the lips. This report is the first to document GA’s histopathological features on the lips, highlighting its potential occurrence in atypical sites.

## Introduction

Glycogenic acanthosis (GA) is a benign condition characterized by asymptomatic white papules or plaques, most commonly found in the lower third of the esophagus.^1^ Its prevalence ranges from 5% to 15%, and it is typically an incidental finding during endoscopy.^2,3^ GA is associated with conditions such as gastroesophageal reflux, Phosphtase and tensin homolog (PTEN) hamartoma tumor syndrome, insulin resistance, and metabolic syndrome.^3,4^ Although some associations with celiac disease in children have been reported, involvement of the lips has not been documented.^3^ This case report describes a rare presentation of GA on the lips, adding to the clinical spectrum of this condition.

## Case presentation

A 55-year-old male presented with asymptomatic white lesions on the upper and lower lips, that had been present for several years. The lesions first appeared on the lower lip and subsequently spread to the upper lip within 2 weeks. The patient noted an increase in the number and size of the lesions but experienced no associated symptoms such as itching or pain. He had not sought any treatment for these lesions.

The patient was a half-pack-per-day smoker with a history of hypertension, prediabetes, and lower back pain but was not on any medications. He was retired and had no family history of skin diseases or early-onset breast or colon cancer. The patient did not present with any features of PTEN hamartoma tumor syndrome. Due to his smoking habit, initial differential diagnoses included leukoplakia, mucosal squamous cell carcinoma (SCC), mucosal lichen planus, and verruca. The patient reported no systemic symptoms upon review.

**Figure 1.**
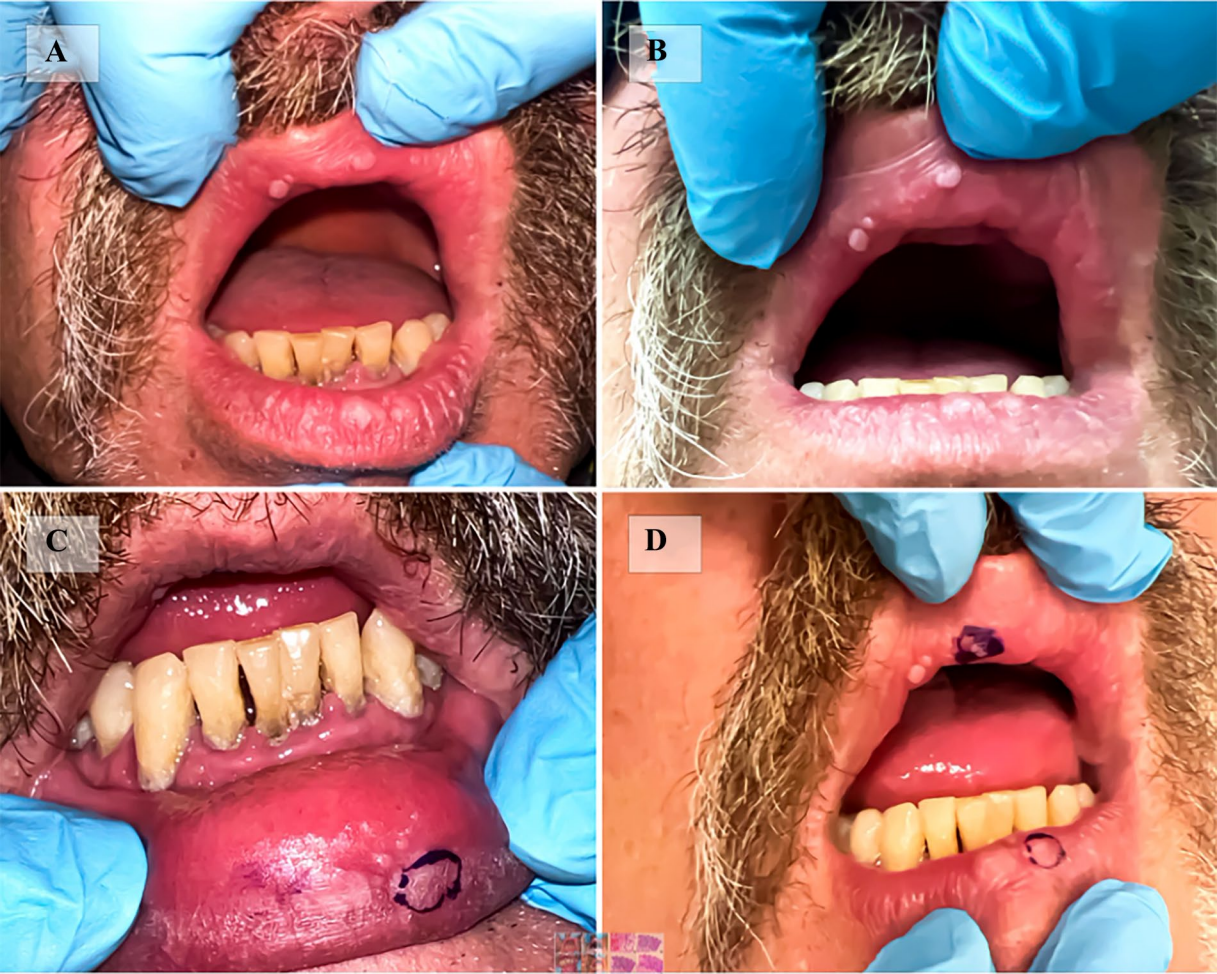
Demonstrates the GA present on both the upper and lower lips of our case. (A,B) Three white 2-6mm acanthotic round papules on the wet-dry vermillion border of the right upper lip (A, with flash, B without flash). (C) Upon stretching the lower lip, a linear arrangement of three 1-2mm white papules are revealed on the mucosal surface. The blue ink marked one biopsy site. (D) The blue ink marked the location of the two punch biopsies that were taken from the upper and lower lip. GA: Glycogenic acanthosis.

On examination, the patient, who had Fitzpatrick skin type II, appeared otherwise healthy. Three white acanthotic round papules, ranging from 2 to 6 mm, were noted on the wet-dry vermilion border of the right upper lip (Figure 1(A) and (B)). The mid-lower lip exhibited two larger papules, 7–8 mm in size, straddling the wet-dry vermilion border (Figure 1(A) and (B)). Upon stretching the lower lip, a linear arrangement of three 1–2 mm papules was observed on the mucosal surface (Figure 1(C) and (D)).

**Figure 2.**
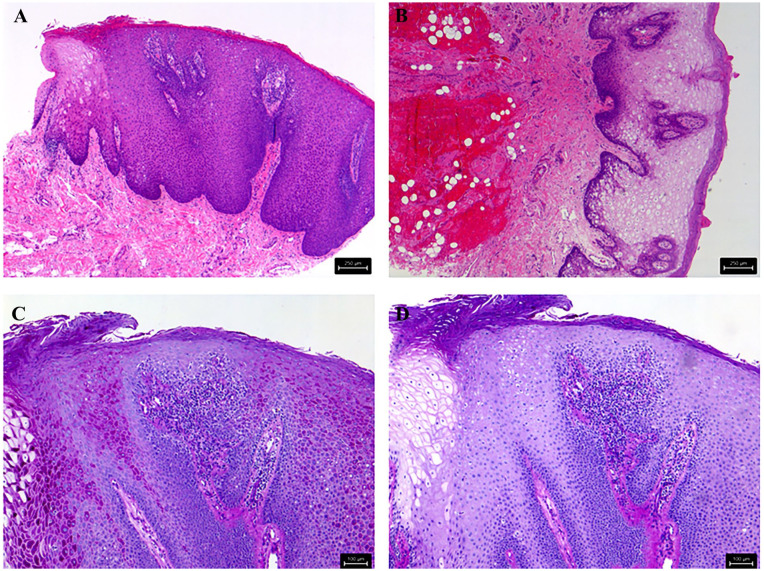
Histopathologically, the lesions showed hyperkeratosis and acanthosis with keratinocytes having abundant clear cytoplasm ((A, B) magnification 50×) containing glycogen as demonstrated by PAS ((C) magnification 100×) and PAS with diastase digestion ((D) magnification 100×). PAS: Periodic acid-Schiff.

Punch biopsies were performed on the papules from both the upper and lower lips. Histopathological examination of these biopsies revealed acanthotic squamous epithelium with basal hyperplasia and hyperkeratosis. The keratinocytes were enlarged with abundant pale to clear cytoplasm, which stained positive with Periodic acid-Schiff and showed sensitivity to diastase confirming the presence of intracytoplasmic glycogen (Figure 2). These findings confirmed the diagnosis of GA.

## Discussion

The histological features observed in the lip lesions of our patient align with those typically described in esophageal GA, a benign condition characterized by thickening of the squamous epithelium and an accumulation of glycogen within epithelial cells.^2^ Initially described by Rywlin and Ortega in 1970, esophageal GA is frequently encountered during endoscopic procedures, particularly in older adults.^5,6^ The occurrence of GA in the oral cavity is rare, with few documented cases, and to our knowledge, this report is the first to document GA on the lips in English literature.^7^ However, there is one report of GA on the lips found in the Japanese literature, further supporting the possibility of this rare manifestation.^8^

GA is generally considered a benign entity, often identified incidentally during endoscopic examinations for other conditions.^9^ It has been associated with conditions such as PTEN hamartoma tumor syndrome, insulin resistance, and metabolic syndrome.^4,10^ Given these associations, we recommended that the patient undergo screening for these conditions through his family physician. Despite these associations, GA itself is not typically indicative of malignancy or severe systemic disease.^10,11^

The pathogenesis of GA remains unclear, but it has been postulated that chronic irritation or inflammation might play a role in its development.^10,12^ In our patient, the chronic irritation from smoking could have contributed to the development of GA on the lips. This aligns with previous reports suggesting that irritation might be a potential cause of GA.^6,9,12^

Treatment of GA is usually not necessary due to its benign nature. We are not aware of any studies showing effective treatment for these lesions, especially when they are usually not found in cosmetically sensitive areas. Potential treatment options may include laser ablation or surgical excision. In this case, the patient was not interested in invasive therapies, so we offered a trial of topical steroids and cryotherapy, although he had not pursued these treatments at the time of this report.

This is the first reported case of GA of the cutaneous and mucosal lips in English literature. Gotur et al.^13^ reported a case of GA of the tongue, and reviewed eight other cases of “extra-esophageal” GA, but none of them included lesions on the lips (sites reported included larynx, gingivae, tongue, buccal mucosa).^13^ Another possible diagnosis is that this patient had a variant of Clear Cell Acanthoma (CCA). While CCA is considered a neoplasm with clonal keratinocytes and a sharply demarcated boundary from normal epithelium, GA is a reactive hyperplasia due to glycogen accumulation. Their histological features are similar, but it remains unclear if they are related. In addition, CCAs are usually found only on the skin, and most often as a red to brown dome-shaped papule on the leg of an elderly person.^14^ Although “Mucosal CCA” is named as a CCA variant,^14^ there have only been two documented cases in the literature of CCA on the mucosa of the lips, only one of which was of a nonpigmented lesion.^15,16^

This case underscores the importance of considering GA in the differential diagnosis of white papules on the lips. Other potential differential diagnoses include white sponge nevus, lichen planus, keratinizing dysplasia, mucosal SCC, leukoplakia, and verruca. A biopsy is essential to exclude these conditions, especially to rule out dysplasia.^17–20^ While traditionally associated with the esophagus, GA can present in atypical sites, expanding the clinical spectrum of this condition.^9,13,21^

The rarity of GA on the lips highlights the need for further research to understand its pathogenesis and potential associations with systemic conditions. Documenting such atypical presentations can aid in the recognition and management of this benign condition in clinical practice.

## Conclusion

This rare presentation of GA on the lips expands the clinical spectrum of this benign lesion and should be considered in the differential diagnosis of white papules on the lips. While GA is traditionally described in the esophagus, this case highlights its potential occurrence in the oral and lip regions. This is the first histopathological documentation of lip involvement in cases of GA in English literature, emphasizing the need to include this diagnosis among the various lesions that present as whitish plaques.
